# [μ-Butane-1,4-diylbis(di­phenyl­phos­phane)-κ^2^ *P*:*P*′]bis­{[butane-1,4-diylbis(di­phenyl­phosphane)-κ^2^ *P*,*P*′]copper(I)} bis­(hexa­fluorido­phosphate) diethyl ether disolvate

**DOI:** 10.1107/S1600536814009763

**Published:** 2014-05-10

**Authors:** Michihiro Nishikawa, Asumi Akiyama, Taro Tsubomura

**Affiliations:** a Department of Materials and Life Science, Seikei University, 3-3-1 Kichijoji-kitamachi, Musashino-shi, Tokyo, Japan

## Abstract

In the centrosymmetric dinuclear copper(I) complex cation of the title compound, [Cu_2_(C_28_H_28_P_2_)_3_](PF_6_)_2_·2C_4_H_10_O, the Cu^I^ atom is bonded to three P atoms of two butane-1,4-diylbis(di­phenyl­phosphane) (dppb) ligands with a triangular coordination geometry. One of these P atoms belongs to a bridging dppb ligand [Cu—P = 2.2381 (5) Å] and two belong to a chelating dppb ligand [Cu—P = 2.2450 (6) and 2.2628 (5) Å]. The bridging dppb ligand lies on an inversion centre. In the crystal, the cation and the PF_6_^−^ anion are linked by C—H⋯F inter­actions, forming a tape along [110]. The cation and the diethyl ether solvent mol­ecule are also linked by a C—H⋯O inter­action.

## Related literature   

For general background to emissive copper(I) complexes, see: McMillin & McNett (1998 ▶). For copper(I) complexes bearing dppb ligands, see: Comba *et al.* (1999 ▶); Kitagawa *et al.* (1995 ▶). For our previous work related to the photophysical properties of copper(I) complexes bearing dppb and di­imine ligands, see: Saito *et al.* (2006 ▶).
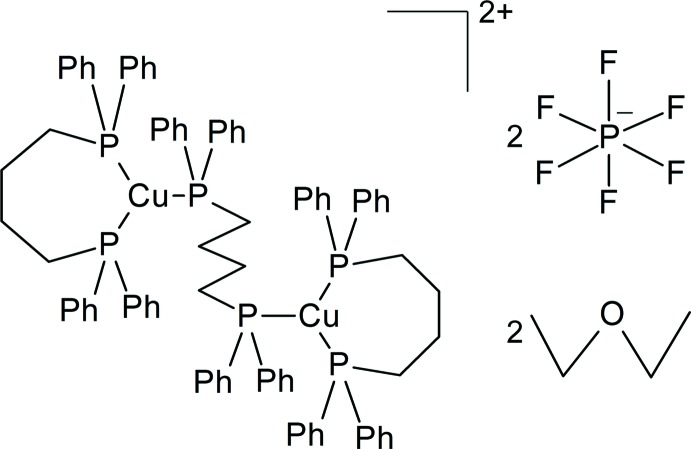


## Experimental   

### 

#### Crystal data  

[Cu_2_(C_28_H_28_P_2_)_3_](PF_6_)_2_·2C_4_H_10_O
*M* *_r_* = 1844.61
Triclinic, 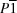

*a* = 12.7912 (13) Å
*b* = 13.7023 (16) Å
*c* = 14.3811 (13) Å
α = 105.595 (3)°
β = 90.858 (2)°
γ = 111.932 (3)°
*V* = 2233.2 (4) Å^3^
*Z* = 1
Mo *K*α radiation
μ = 0.69 mm^−1^
*T* = 123 K
0.5 × 0.5 × 0.4 mm

#### Data collection  

Rigaku Saturn70 CCD diffractometer
Absorption correction: multi-scan (*REQAB*; Rigaku, 1998 ▶) *T*_min_ = 0.687, *T*_max_ = 0.758
20774 measured reflections
9436 independent reflections
8266 reflections with *I* > 2σ(*I*)
*R*_int_ = 0.024

#### Refinement  

*R*[*F* ^2^ > 2σ(*F* ^2^)] = 0.036
*wR*(*F* ^2^) = 0.090
*S* = 1.06
9436 reflections
523 parameters
H-atom parameters constrained
Δρ_max_ = 0.41 e Å^−3^
Δρ_min_ = −0.35 e Å^−3^

### 

Data collection: *CrystalClear* (Rigaku, 2000 ▶); cell refinement: *CrystalClear*; data reduction: *CrystalClear*; program(s) used to solve structure: *SIR92* (Altomare *et al.*, 1993 ▶); program(s) used to refine structure: *SHELXL97* (Sheldrick, 2008 ▶); molecular graphics: *ORTEP-3 for Windows* (Farrugia, 2012 ▶); software used to prepare material for publication: *publCIF* (Westrip, 2010 ▶).

## Supplementary Material

Crystal structure: contains datablock(s) global, I. DOI: 10.1107/S1600536814009763/is5355sup1.cif

Structure factors: contains datablock(s) I. DOI: 10.1107/S1600536814009763/is5355Isup2.hkl

CCDC reference: 1000315

Additional supporting information:  crystallographic information; 3D view; checkCIF report

## Figures and Tables

**Table 1:** Hydrogen-bond geometry (Å, °)

*D*—H⋯*A*	*D*—H	H⋯*A*	*D*⋯*A*	*D*—H⋯*A*
C016—H016⋯F006	0.95	2.47	3.318 (3)	149
C018—H018⋯F008^i^	0.95	2.53	3.286 (3)	137
C043—H04*C*⋯F006	0.99	2.45	3.345 (3)	150
C043—H04*C*⋯F009	0.99	2.53	3.458 (3)	156
C049—H049⋯O012^ii^	0.95	2.46	3.393 (3)	169

Symmetry codes: (i) 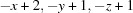; (ii) 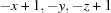.

**Fig. 1.**
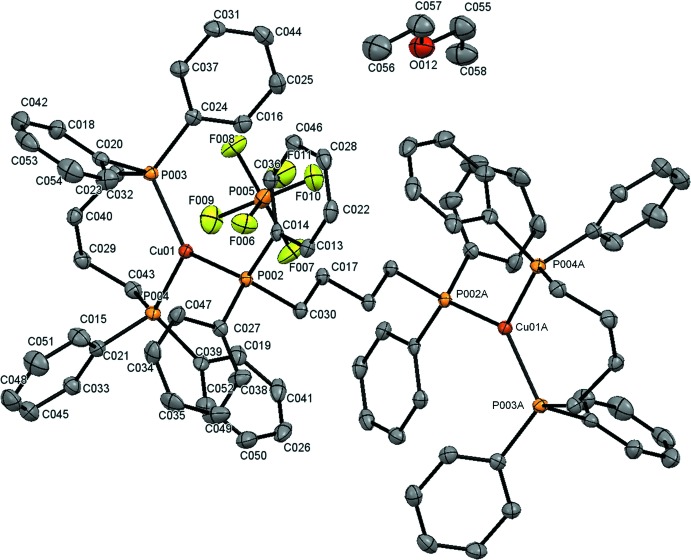
ORTEP drawing of [Cu2(µ-dppb)(dppb)2](PF6)2·2Et2O, showing 50% probability displacement ellipsoids. Hydrogen atoms are omitted for clarity. Symmetry code (A); –x + 1, –y, –z + 1.

## supplementary crystallographic information

### 1. Comment

Copper(I) complexes bearing diphosphane ligands are of much interest for luminescence devices (McMillin & McNett, 1998) and catalysts. The spectroscopic study for the coordination compounds of copper(I) ion and 1,4-bis(diphenylphosphino)butane(dppb) ligand has been reported (Comba *et al.*, 1999). The crystal structure of a copper(I) complex bearing dppb, such as [Cu_2_(dppb)_2_(ClO_4_)_2_] where two copper atoms are bridged by two dppb unit, has been reported (Kitagawa *et al.*, 1995). We have reported the crystal structure of an emissive dinuclear copper(I) complex bearing dppb and diimine ligands (Saito *et al.*, 2006), in which the copper atoms are also bridged by two dppb ligands.

We describe herein the structure of a dinuclear copper(I) complex cation bearing two types of dppb ligands; one is a bridging ligand which connects two copper atoms using two phosphorus atoms, and the other is the chelating ligand which binds one copper atom using two phosphorus atoms. In other words, two copper atoms in the complex are bridged by one dppb ligand. The centre of inversion lies on the bridging dppb ligand. The asymmetric unit consists of a half of the complex cation, a PF_6_ anion and a diethylether solvent molecule (Fig. 1). Each copper atom is connected by three phosphorus atoms with a triangle coordination geometry. The bond length between copper and phosphorus atom of the bridging dppb ligand is Cu—P = 2.2381 (5) Å, and those between copper and phosphorus atoms of the chelating ligands are Cu—P = 2.2450 (6) and 2.2628 (5) Å. This finding is useful for strategy for creation of characteristic dinuclear copper(I) complexes which exhibit unique properties.

### 2. Experimental

Under an argon atmosphere, [Cu(MeCN)_4_]PF_6_ (75 mg, 0.20 mmol) was added to dppb (82 mg, 0.30 mmol) in a 5 mL dichloromethane. The reaction mixture was stirred for 30 min at room temperature. Diethyl ether was added to the solution to precipitate the product as a white solid, which was filtered and washed with diethyl ether: yield, 126 mg (0.162 mmol, 81%). Elemental Analysis Calcd. for C_84_H_84_F_12_P_8_Cu_2_: C 59.47, H 4.99, found C 58.58, H 4.92. Single crystals suitable for X-ray diffraction were obtained by slow diffusion of diethylether in a dichloromethane solution of the complex.

### 3. Refinement

All H atoms were positioned geometrically and refined using a riding model, with C—H = 0.99 Å and *U*_iso_(H) = 1.2*U*_eq_(C) for methylene groups, C—H = 0.98 Å and *U*_iso_(H) = 1.5*U*_eq_(C) for methyl groups, and C—H = 0.95 Å and *U*_iso_(H) = 1.2*U*_eq_(C) for aromatic groups.

### Crystal data

**Table:**

[Cu_2_(C_28_H_28_P_2_)_3_](PF_6_)_2_·2C_4_H_10_O	*Z* = 1
*M**_r_* = 1844.61	*F*(000) = 958
Triclinic, *P*1	*D*_x_ = 1.372 Mg m^−^^3^
Hall symbol: -P 1	Mo *K*α radiation, λ = 0.7107 Å
*a* = 12.7912 (13) Å	Cell parameters from 5352 reflections
*b* = 13.7023 (16) Å	θ = 3.1–27.5°
*c* = 14.3811 (13) Å	µ = 0.69 mm^−^^1^
α = 105.595 (3)°	*T* = 123 K
β = 90.858 (2)°	Block, colorless
γ = 111.932 (3)°	0.5 × 0.5 × 0.4 mm
*V* = 2233.2 (4) Å^3^	

### Data collection

**Table:**

Rigaku Saturn70 CCD diffractometer	9436 independent reflections
Graphite monochromator	8266 reflections with *I* > 2σ(*I*)
Detector resolution: 28.5714 pixels mm^-1^	*R*_int_ = 0.024
ω scans	θ_max_ = 27.5°, θ_min_ = 3.1°
Absorption correction: multi-scan (*REQAB*; Rigaku, 1998)	*h* = −16→16
*T*_min_ = 0.687, *T*_max_ = 0.758	*k* = −17→17
20774 measured reflections	*l* = −18→18

### Refinement

**Table:**

Refinement on *F*^2^	Primary atom site location: structure-invariant direct methods
Least-squares matrix: full	Secondary atom site location: difference Fourier map
*R*[*F*^2^ > 2σ(*F*^2^)] = 0.036	Hydrogen site location: inferred from neighbouring sites
*wR*(*F*^2^) = 0.090	H-atom parameters constrained
*S* = 1.06	*w* = 1/[σ^2^(*F*_o_^2^) + (0.0437*P*)^2^ + 1.0988*P*] where *P* = (*F*_o_^2^ + 2*F*_c_^2^)/3
9436 reflections	(Δ/σ)_max_ = 0.001
523 parameters	Δρ_max_ = 0.41 e Å^−^^3^
0 restraints	Δρ_min_ = −0.35 e Å^−^^3^

### Special details

**Table:**

Geometry. All e.s.d.'s (except the e.s.d. in the dihedral angle between two l.s. planes) are estimated using the full covariance matrix. The cell e.s.d.'s are taken into account individually in the estimation of e.s.d.'s in distances, angles and torsion angles; correlations between e.s.d.'s in cell parameters are only used when they are defined by crystal symmetry. An approximate (isotropic) treatment of cell e.s.d.'s is used for estimating e.s.d.'s involving l.s. planes.
Refinement. Refinement of *F*^2^ against ALL reflections. The weighted *R*-factor *wR* and goodness of fit *S* are based on *F*^2^, conventional *R*-factors *R* are based on *F*, with *F* set to zero for negative *F*^2^. The threshold expression of *F*^2^ > σ(*F*^2^) is used only for calculating *R*-factors(gt) *etc*. and is not relevant to the choice of reflections for refinement. *R*-factors based on *F*^2^ are statistically about twice as large as those based on *F*, and *R*- factors based on ALL data will be even larger.

### Fractional atomic coordinates and isotropic or equivalent isotropic displacement parameters (Å^2^)

**Table:**

	*x*	*y*	*z*	*U*_iso_*/*U*_eq_	
Cu01	0.736171 (18)	0.110853 (17)	0.302039 (16)	0.01521 (7)	
P002	0.54853 (4)	0.01284 (4)	0.26739 (3)	0.01428 (10)	
P003	0.84597 (4)	0.28839 (4)	0.32139 (3)	0.01596 (10)	
P004	0.83982 (4)	0.01424 (4)	0.32330 (4)	0.01677 (11)	
P005	0.86536 (5)	0.27004 (4)	0.66681 (4)	0.02525 (12)	
F006	0.81271 (11)	0.20082 (10)	0.55514 (9)	0.0341 (3)	
F007	0.81659 (13)	0.15823 (12)	0.69569 (12)	0.0501 (4)	
F008	0.91334 (11)	0.37992 (10)	0.63536 (10)	0.0366 (3)	
F009	0.98020 (11)	0.25176 (12)	0.64561 (11)	0.0425 (3)	
F010	0.74923 (13)	0.28665 (13)	0.68407 (12)	0.0511 (4)	
F011	0.91859 (15)	0.33750 (13)	0.77641 (10)	0.0540 (4)	
O012	0.73139 (16)	0.56078 (14)	0.97322 (12)	0.0419 (4)	
C013	0.34145 (16)	0.02452 (16)	0.21426 (14)	0.0196 (4)	
H013	0.3106	−0.0537	0.1947	0.023*	
C014	0.45566 (15)	0.08317 (15)	0.25249 (13)	0.0158 (4)	
C015	0.8519 (2)	−0.04572 (18)	0.12452 (15)	0.0298 (5)	
H015	0.8053	−0.0064	0.1211	0.036*	
C016	0.74237 (17)	0.37377 (16)	0.47064 (14)	0.0222 (4)	
H016	0.7317	0.3085	0.4875	0.027*	
C017	0.52540 (15)	0.02814 (15)	0.46177 (12)	0.0164 (4)	
H01A	0.4992	0.0882	0.4644	0.02*	
H01B	0.6093	0.0611	0.4763	0.02*	
C018	0.96731 (19)	0.38988 (17)	0.18627 (15)	0.0269 (4)	
H018	1.027	0.4346	0.2381	0.032*	
C019	0.75556 (17)	−0.07541 (18)	0.47393 (15)	0.0252 (4)	
H019	0.8023	−0.0054	0.5171	0.03*	
C020	0.86868 (17)	0.31242 (15)	0.20336 (14)	0.0198 (4)	
C021	0.88492 (17)	−0.05330 (15)	0.21397 (14)	0.0204 (4)	
C022	0.27251 (17)	0.08035 (18)	0.20464 (15)	0.0250 (4)	
H022	0.1948	0.0401	0.1782	0.03*	
C023	0.7826 (2)	0.24778 (18)	0.12597 (15)	0.0295 (5)	
H023	0.7147	0.1943	0.1365	0.035*	
C024	0.79829 (15)	0.39306 (15)	0.39064 (13)	0.0171 (4)	
C025	0.70245 (18)	0.44887 (17)	0.52550 (15)	0.0265 (4)	
H025	0.6657	0.4357	0.5805	0.032*	
C026	0.61412 (19)	−0.25764 (19)	0.44828 (19)	0.0346 (5)	
H026	0.564	−0.3124	0.4737	0.042*	
C027	0.51341 (16)	−0.09941 (15)	0.15446 (13)	0.0173 (4)	
C028	0.31688 (18)	0.19436 (18)	0.23353 (15)	0.0257 (4)	
H028	0.2697	0.2322	0.2266	0.031*	
C029	1.06889 (16)	0.17199 (16)	0.37292 (15)	0.0228 (4)	
H02A	1.1016	0.1267	0.3269	0.027*	
H02B	1.1285	0.2195	0.4288	0.027*	
C030	0.49050 (16)	−0.05367 (15)	0.35987 (13)	0.0166 (4)	
H03A	0.4065	−0.0877	0.3459	0.02*	
H03B	0.5178	−0.113	0.3571	0.02*	
C031	0.77138 (19)	0.56291 (17)	0.42141 (16)	0.0289 (5)	
H031	0.7812	0.6279	0.4045	0.035*	
C032	0.98833 (16)	0.32387 (16)	0.38117 (14)	0.0196 (4)	
H03C	1.0367	0.4014	0.3865	0.024*	
H03D	0.9833	0.3164	0.4477	0.024*	
C033	0.95356 (17)	−0.11111 (16)	0.21745 (15)	0.0241 (4)	
H033	0.9766	−0.1171	0.2781	0.029*	
C034	0.53314 (19)	−0.15555 (18)	−0.01643 (15)	0.0293 (5)	
H034	0.5591	−0.1366	−0.0732	0.035*	
C035	0.4782 (2)	−0.26468 (18)	−0.02033 (15)	0.0327 (5)	
H035	0.4677	−0.321	−0.0794	0.039*	
C036	0.49914 (16)	0.19781 (16)	0.28186 (13)	0.0193 (4)	
H036	0.5767	0.2384	0.3085	0.023*	
C037	0.81278 (17)	0.48876 (16)	0.36653 (15)	0.0237 (4)	
H037	0.851	0.5032	0.3125	0.028*	
C038	0.4563 (2)	−0.21002 (17)	0.14908 (15)	0.0303 (5)	
H038	0.4295	−0.2294	0.2055	0.036*	
C039	0.75975 (16)	−0.09669 (16)	0.37369 (15)	0.0205 (4)	
C040	1.04248 (16)	0.24690 (16)	0.32190 (15)	0.0214 (4)	
H04A	0.9906	0.1996	0.2612	0.026*	
H04B	1.1141	0.2931	0.3031	0.026*	
C041	0.68313 (18)	−0.1564 (2)	0.51040 (17)	0.0310 (5)	
H041	0.6811	−0.142	0.5786	0.037*	
C042	0.9791 (2)	0.40231 (19)	0.09362 (17)	0.0367 (6)	
H042	1.047	0.4552	0.0823	0.044*	
C043	0.96989 (16)	0.09423 (16)	0.41050 (14)	0.0211 (4)	
H04C	0.951	0.1384	0.4693	0.025*	
H04D	0.9956	0.0422	0.4304	0.025*	
C044	0.71597 (18)	0.54341 (18)	0.50043 (16)	0.0286 (5)	
H044	0.6873	0.5944	0.5373	0.034*	
C045	0.98856 (19)	−0.15988 (18)	0.13317 (17)	0.0314 (5)	
H045	1.0358	−0.1987	0.1364	0.038*	
C046	0.42993 (18)	0.25338 (17)	0.27254 (14)	0.0236 (4)	
H046	0.4602	0.3316	0.2929	0.028*	
C047	0.55046 (18)	−0.07349 (17)	0.07042 (14)	0.0253 (4)	
H047	0.5882	0.0015	0.0725	0.03*	
C048	0.9550 (2)	−0.1523 (2)	0.04439 (17)	0.0380 (6)	
H048	0.9787	−0.1862	−0.0134	0.046*	
C049	0.4382 (2)	−0.29210 (19)	0.06203 (17)	0.0392 (6)	
H049	0.3983	−0.3672	0.0589	0.047*	
C050	0.6176 (2)	−0.27972 (18)	0.34889 (19)	0.0356 (5)	
H050	0.5701	−0.3497	0.3062	0.043*	
C051	0.8868 (2)	−0.0953 (2)	0.04013 (17)	0.0415 (6)	
H051	0.8636	−0.0899	−0.0207	0.05*	
C052	0.69051 (18)	−0.19939 (17)	0.31144 (17)	0.0276 (5)	
H052	0.693	−0.2148	0.2432	0.033*	
C053	0.8923 (2)	0.3379 (2)	0.01796 (16)	0.0379 (6)	
H053	0.9003	0.3473	−0.045	0.046*	
C054	0.7946 (2)	0.2605 (2)	0.03378 (16)	0.0382 (6)	
H054	0.7354	0.2156	−0.0184	0.046*	
C055	0.7128 (3)	0.6010 (2)	1.07002 (19)	0.0475 (7)	
H05A	0.6304	0.5702	1.0752	0.057*	
H05B	0.7396	0.6822	1.0887	0.057*	
C056	0.6958 (3)	0.5383 (3)	0.8049 (2)	0.0558 (8)	
H05C	0.6541	0.5551	0.7578	0.084*	
H05D	0.6711	0.4583	0.7899	0.084*	
H05E	0.7775	0.5713	0.8011	0.084*	
C057	0.6727 (3)	0.5841 (2)	0.90481 (19)	0.0464 (6)	
H05F	0.6968	0.6649	0.9198	0.056*	
H05G	0.5901	0.5517	0.9085	0.056*	
C058	0.7755 (3)	0.5687 (2)	1.1374 (2)	0.0533 (7)	
H05H	0.7622	0.5966	1.2044	0.08*	
H05I	0.8571	0.6001	1.1327	0.08*	
H05J	0.7483	0.4882	1.1191	0.08*	

### Atomic displacement parameters (Å^2^)

**Table:**

	*U*^11^	*U*^22^	*U*^33^	*U*^12^	*U*^13^	*U*^23^
Cu01	0.01457 (12)	0.01581 (12)	0.01666 (12)	0.00650 (9)	0.00247 (8)	0.00621 (9)
P002	0.0150 (2)	0.0165 (2)	0.0123 (2)	0.00630 (19)	0.00294 (17)	0.00560 (17)
P003	0.0153 (2)	0.0153 (2)	0.0180 (2)	0.00644 (19)	0.00140 (18)	0.00544 (18)
P004	0.0173 (2)	0.0177 (2)	0.0192 (2)	0.00926 (19)	0.00360 (18)	0.00798 (19)
P005	0.0228 (3)	0.0251 (3)	0.0236 (3)	0.0035 (2)	0.0038 (2)	0.0090 (2)
F006	0.0344 (7)	0.0299 (7)	0.0327 (7)	0.0125 (6)	−0.0080 (6)	0.0017 (5)
F007	0.0513 (9)	0.0383 (8)	0.0586 (10)	0.0030 (7)	0.0066 (7)	0.0320 (7)
F008	0.0379 (8)	0.0276 (7)	0.0348 (7)	0.0002 (6)	−0.0031 (6)	0.0130 (6)
F009	0.0270 (7)	0.0575 (9)	0.0471 (8)	0.0182 (7)	0.0010 (6)	0.0200 (7)
F010	0.0401 (8)	0.0523 (9)	0.0642 (10)	0.0218 (7)	0.0275 (8)	0.0160 (8)
F011	0.0779 (12)	0.0463 (9)	0.0216 (7)	0.0082 (8)	−0.0026 (7)	0.0081 (6)
O012	0.0539 (11)	0.0360 (9)	0.0362 (9)	0.0183 (8)	0.0060 (8)	0.0104 (7)
C013	0.0174 (9)	0.0223 (9)	0.0193 (9)	0.0061 (8)	0.0041 (7)	0.0090 (8)
C014	0.0172 (9)	0.0218 (9)	0.0113 (8)	0.0084 (8)	0.0057 (7)	0.0082 (7)
C015	0.0411 (13)	0.0323 (11)	0.0246 (11)	0.0223 (10)	0.0048 (9)	0.0107 (9)
C016	0.0250 (10)	0.0217 (10)	0.0197 (10)	0.0084 (8)	0.0014 (8)	0.0067 (8)
C017	0.0169 (9)	0.0200 (9)	0.0137 (9)	0.0075 (7)	0.0036 (7)	0.0065 (7)
C018	0.0327 (12)	0.0247 (10)	0.0237 (10)	0.0101 (9)	0.0090 (9)	0.0090 (8)
C019	0.0233 (10)	0.0330 (11)	0.0289 (11)	0.0168 (9)	0.0070 (8)	0.0160 (9)
C020	0.0268 (10)	0.0170 (9)	0.0192 (9)	0.0126 (8)	0.0038 (8)	0.0050 (7)
C021	0.0220 (10)	0.0183 (9)	0.0209 (9)	0.0074 (8)	0.0043 (8)	0.0066 (7)
C022	0.0159 (9)	0.0374 (12)	0.0254 (10)	0.0110 (9)	0.0052 (8)	0.0141 (9)
C023	0.0336 (12)	0.0300 (11)	0.0236 (11)	0.0117 (10)	−0.0012 (9)	0.0071 (9)
C024	0.0135 (9)	0.0183 (9)	0.0177 (9)	0.0058 (7)	−0.0020 (7)	0.0033 (7)
C025	0.0251 (11)	0.0317 (11)	0.0214 (10)	0.0113 (9)	0.0054 (8)	0.0054 (9)
C026	0.0294 (12)	0.0351 (12)	0.0573 (16)	0.0185 (10)	0.0195 (11)	0.0326 (12)
C027	0.0192 (9)	0.0202 (9)	0.0144 (9)	0.0098 (8)	0.0018 (7)	0.0050 (7)
C028	0.0266 (11)	0.0379 (12)	0.0256 (11)	0.0215 (10)	0.0111 (8)	0.0169 (9)
C029	0.0179 (9)	0.0253 (10)	0.0281 (11)	0.0116 (8)	0.0025 (8)	0.0077 (8)
C030	0.0181 (9)	0.0187 (9)	0.0139 (9)	0.0072 (7)	0.0027 (7)	0.0062 (7)
C031	0.0348 (12)	0.0238 (10)	0.0347 (12)	0.0172 (10)	0.0054 (9)	0.0105 (9)
C032	0.0167 (9)	0.0207 (9)	0.0227 (10)	0.0082 (8)	0.0016 (7)	0.0073 (8)
C033	0.0256 (10)	0.0250 (10)	0.0248 (10)	0.0131 (9)	0.0046 (8)	0.0077 (8)
C034	0.0322 (12)	0.0371 (12)	0.0151 (10)	0.0110 (10)	0.0079 (8)	0.0058 (9)
C035	0.0479 (14)	0.0306 (11)	0.0182 (10)	0.0205 (11)	0.0014 (9)	−0.0019 (9)
C036	0.0180 (9)	0.0232 (10)	0.0164 (9)	0.0076 (8)	0.0030 (7)	0.0060 (7)
C037	0.0260 (10)	0.0222 (10)	0.0260 (10)	0.0113 (9)	0.0051 (8)	0.0094 (8)
C038	0.0475 (14)	0.0224 (10)	0.0190 (10)	0.0097 (10)	0.0038 (9)	0.0087 (8)
C039	0.0200 (10)	0.0226 (10)	0.0284 (10)	0.0138 (8)	0.0077 (8)	0.0142 (8)
C040	0.0166 (9)	0.0238 (10)	0.0260 (10)	0.0081 (8)	0.0049 (8)	0.0104 (8)
C041	0.0273 (11)	0.0471 (14)	0.0375 (13)	0.0241 (11)	0.0152 (10)	0.0282 (11)
C042	0.0527 (15)	0.0316 (12)	0.0366 (13)	0.0213 (12)	0.0243 (12)	0.0192 (10)
C043	0.0209 (10)	0.0241 (10)	0.0209 (10)	0.0115 (8)	0.0017 (8)	0.0070 (8)
C044	0.0261 (11)	0.0292 (11)	0.0301 (11)	0.0161 (9)	0.0022 (9)	0.0010 (9)
C045	0.0335 (12)	0.0292 (11)	0.0352 (12)	0.0176 (10)	0.0106 (10)	0.0076 (9)
C046	0.0294 (11)	0.0237 (10)	0.0231 (10)	0.0149 (9)	0.0073 (8)	0.0088 (8)
C047	0.0258 (11)	0.0248 (10)	0.0201 (10)	0.0033 (9)	0.0063 (8)	0.0079 (8)
C048	0.0525 (15)	0.0363 (13)	0.0290 (12)	0.0233 (12)	0.0167 (11)	0.0064 (10)
C049	0.0653 (17)	0.0211 (11)	0.0265 (12)	0.0130 (11)	0.0015 (11)	0.0057 (9)
C050	0.0327 (12)	0.0226 (11)	0.0552 (16)	0.0113 (10)	0.0122 (11)	0.0164 (11)
C051	0.0646 (17)	0.0483 (15)	0.0198 (11)	0.0307 (14)	0.0071 (11)	0.0102 (10)
C052	0.0313 (11)	0.0231 (10)	0.0327 (12)	0.0136 (9)	0.0082 (9)	0.0106 (9)
C053	0.0695 (18)	0.0430 (14)	0.0214 (11)	0.0405 (14)	0.0166 (11)	0.0141 (10)
C054	0.0543 (16)	0.0433 (14)	0.0196 (11)	0.0258 (13)	−0.0025 (10)	0.0041 (10)
C055	0.0595 (18)	0.0430 (15)	0.0371 (14)	0.0219 (14)	0.0069 (12)	0.0050 (12)
C056	0.080 (2)	0.0540 (17)	0.0420 (16)	0.0326 (17)	0.0099 (15)	0.0193 (13)
C057	0.0589 (17)	0.0430 (15)	0.0440 (15)	0.0261 (14)	0.0047 (13)	0.0145 (12)
C058	0.080 (2)	0.0392 (15)	0.0381 (15)	0.0245 (15)	0.0001 (14)	0.0071 (12)

### Geometric parameters (Å, º)

**Table:**

Cu01—P002	2.2381 (5)	C029—H02A	0.99
Cu01—P003	2.2450 (6)	C029—H02B	0.99
Cu01—P004	2.2628 (5)	C030—H03A	0.99
P002—C027	1.8232 (18)	C030—H03B	0.99
P002—C030	1.8239 (18)	C031—C044	1.382 (3)
P002—C014	1.8287 (19)	C031—C037	1.385 (3)
P003—C020	1.821 (2)	C031—H031	0.95
P003—C024	1.8233 (19)	C032—C040	1.549 (3)
P003—C032	1.8326 (19)	C032—H03C	0.99
P004—C021	1.8179 (19)	C032—H03D	0.99
P004—C039	1.827 (2)	C033—C045	1.386 (3)
P004—C043	1.838 (2)	C033—H033	0.95
P005—F011	1.5865 (15)	C034—C035	1.379 (3)
P005—F008	1.5914 (13)	C034—C047	1.387 (3)
P005—F007	1.5952 (14)	C034—H034	0.95
P005—F010	1.5979 (15)	C035—C049	1.385 (3)
P005—F009	1.6001 (14)	C035—H035	0.95
P005—F006	1.6136 (13)	C036—C046	1.391 (3)
O012—C057	1.400 (3)	C036—H036	0.95
O012—C055	1.412 (3)	C037—H037	0.95
C013—C022	1.393 (3)	C038—C049	1.388 (3)
C013—C014	1.394 (3)	C038—H038	0.95
C013—H013	0.95	C039—C052	1.391 (3)
C014—C036	1.393 (3)	C040—H04A	0.99
C015—C051	1.390 (3)	C040—H04B	0.99
C015—C021	1.390 (3)	C041—H041	0.95
C015—H015	0.95	C042—C053	1.382 (4)
C016—C025	1.382 (3)	C042—H042	0.95
C016—C024	1.397 (3)	C043—H04C	0.99
C016—H016	0.95	C043—H04D	0.99
C017—C030	1.525 (2)	C044—H044	0.95
C017—C017^i^	1.526 (4)	C045—C048	1.383 (3)
C017—H01A	0.99	C045—H045	0.95
C017—H01B	0.99	C046—H046	0.95
C018—C020	1.388 (3)	C047—H047	0.95
C018—C042	1.391 (3)	C048—C051	1.382 (4)
C018—H018	0.95	C048—H048	0.95
C019—C041	1.388 (3)	C049—H049	0.95
C019—C039	1.398 (3)	C050—C052	1.392 (3)
C019—H019	0.95	C050—H050	0.95
C020—C023	1.393 (3)	C051—H051	0.95
C021—C033	1.393 (3)	C052—H052	0.95
C022—C028	1.384 (3)	C053—C054	1.374 (4)
C022—H022	0.95	C053—H053	0.95
C023—C054	1.386 (3)	C054—H054	0.95
C023—H023	0.95	C055—C058	1.501 (4)
C024—C037	1.393 (3)	C055—H05A	0.99
C025—C044	1.387 (3)	C055—H05B	0.99
C025—H025	0.95	C056—C057	1.488 (4)
C026—C041	1.377 (3)	C056—H05C	0.98
C026—C050	1.384 (4)	C056—H05D	0.98
C026—H026	0.95	C056—H05E	0.98
C027—C047	1.391 (3)	C057—H05F	0.99
C027—C038	1.395 (3)	C057—H05G	0.99
C028—C046	1.385 (3)	C058—H05H	0.98
C028—H028	0.95	C058—H05I	0.98
C029—C043	1.534 (3)	C058—H05J	0.98
C029—C040	1.537 (3)		
			
P002—Cu01—P003	133.28 (2)	C040—C032—P003	110.13 (13)
P002—Cu01—P004	114.80 (2)	C040—C032—H03C	109.6
P003—Cu01—P004	111.92 (2)	P003—C032—H03C	109.6
C027—P002—C030	105.01 (8)	C040—C032—H03D	109.6
C027—P002—C014	104.23 (8)	P003—C032—H03D	109.6
C030—P002—C014	103.78 (8)	H03C—C032—H03D	108.1
C027—P002—Cu01	111.49 (6)	C045—C033—C021	120.5 (2)
C030—P002—Cu01	112.37 (6)	C045—C033—H033	119.7
C014—P002—Cu01	118.73 (6)	C021—C033—H033	119.7
C020—P003—C024	106.26 (8)	C035—C034—C047	120.0 (2)
C020—P003—C032	104.91 (9)	C035—C034—H034	120
C024—P003—C032	105.67 (8)	C047—C034—H034	120
C020—P003—Cu01	110.32 (6)	C034—C035—C049	119.93 (19)
C024—P003—Cu01	118.20 (6)	C034—C035—H035	120
C032—P003—Cu01	110.57 (6)	C049—C035—H035	120
C021—P004—C039	104.70 (9)	C046—C036—C014	120.56 (18)
C021—P004—C043	105.34 (9)	C046—C036—H036	119.7
C039—P004—C043	103.73 (9)	C014—C036—H036	119.7
C021—P004—Cu01	115.92 (7)	C031—C037—C024	119.88 (19)
C039—P004—Cu01	111.21 (6)	C031—C037—H037	120.1
C043—P004—Cu01	114.76 (7)	C024—C037—H037	120.1
F011—P005—F008	90.76 (8)	C049—C038—C027	120.6 (2)
F011—P005—F007	90.56 (9)	C049—C038—H038	119.7
F008—P005—F007	178.65 (8)	C027—C038—H038	119.7
F011—P005—F010	91.82 (10)	C052—C039—C019	119.30 (19)
F008—P005—F010	89.62 (8)	C052—C039—P004	119.75 (16)
F007—P005—F010	90.62 (9)	C019—C039—P004	120.41 (16)
F011—P005—F009	90.11 (9)	C029—C040—C032	116.42 (16)
F008—P005—F009	90.01 (8)	C029—C040—H04A	108.2
F007—P005—F009	89.70 (9)	C032—C040—H04A	108.2
F010—P005—F009	178.04 (9)	C029—C040—H04B	108.2
F011—P005—F006	179.17 (9)	C032—C040—H04B	108.2
F008—P005—F006	89.42 (7)	H04A—C040—H04B	107.3
F007—P005—F006	89.25 (8)	C026—C041—C019	120.4 (2)
F010—P005—F006	88.99 (8)	C026—C041—H041	119.8
F009—P005—F006	89.08 (7)	C019—C041—H041	119.8
C057—O012—C055	113.7 (2)	C053—C042—C018	120.2 (2)
C022—C013—C014	120.15 (18)	C053—C042—H042	119.9
C022—C013—H013	119.9	C018—C042—H042	119.9
C014—C013—H013	119.9	C029—C043—P004	115.32 (14)
C036—C014—C013	119.15 (17)	C029—C043—H04C	108.4
C036—C014—P002	119.39 (14)	P004—C043—H04C	108.4
C013—C014—P002	121.44 (14)	C029—C043—H04D	108.4
C051—C015—C021	120.4 (2)	P004—C043—H04D	108.4
C051—C015—H015	119.8	H04C—C043—H04D	107.5
C021—C015—H015	119.8	C031—C044—C025	119.59 (19)
C025—C016—C024	120.52 (19)	C031—C044—H044	120.2
C025—C016—H016	119.7	C025—C044—H044	120.2
C024—C016—H016	119.7	C048—C045—C033	120.3 (2)
C030—C017—C017^i^	111.06 (19)	C048—C045—H045	119.8
C030—C017—H01A	109.4	C033—C045—H045	119.8
C017^i^—C017—H01A	109.4	C028—C046—C036	119.84 (19)
C030—C017—H01B	109.4	C028—C046—H046	120.1
C017^i^—C017—H01B	109.4	C036—C046—H046	120.1
H01A—C017—H01B	108	C034—C047—C027	120.96 (19)
C020—C018—C042	120.3 (2)	C034—C047—H047	119.5
C020—C018—H018	119.8	C027—C047—H047	119.5
C042—C018—H018	119.8	C051—C048—C045	119.6 (2)
C041—C019—C039	120.0 (2)	C051—C048—H048	120.2
C041—C019—H019	120	C045—C048—H048	120.2
C039—C019—H019	120	C035—C049—C038	120.1 (2)
C018—C020—C023	118.60 (19)	C035—C049—H049	120
C018—C020—P003	123.66 (15)	C038—C049—H049	120
C023—C020—P003	117.73 (16)	C026—C050—C052	120.0 (2)
C015—C021—C033	118.84 (18)	C026—C050—H050	120
C015—C021—P004	119.39 (15)	C052—C050—H050	120
C033—C021—P004	121.76 (15)	C048—C051—C015	120.3 (2)
C028—C022—C013	120.17 (19)	C048—C051—H051	119.9
C028—C022—H022	119.9	C015—C051—H051	119.9
C013—C022—H022	119.9	C039—C052—C050	120.1 (2)
C054—C023—C020	120.9 (2)	C039—C052—H052	119.9
C054—C023—H023	119.5	C050—C052—H052	119.9
C020—C023—H023	119.5	C054—C053—C042	120.0 (2)
C037—C024—C016	119.11 (17)	C054—C053—H053	120
C037—C024—P003	123.42 (15)	C042—C053—H053	120
C016—C024—P003	117.45 (14)	C053—C054—C023	119.9 (2)
C016—C025—C044	120.1 (2)	C053—C054—H054	120
C016—C025—H025	120	C023—C054—H054	120
C044—C025—H025	120	O012—C055—C058	109.8 (2)
C041—C026—C050	120.1 (2)	O012—C055—H05A	109.7
C041—C026—H026	119.9	C058—C055—H05A	109.7
C050—C026—H026	119.9	O012—C055—H05B	109.7
C047—C027—C038	118.45 (18)	C058—C055—H05B	109.7
C047—C027—P002	118.10 (15)	H05A—C055—H05B	108.2
C038—C027—P002	123.38 (15)	C057—C056—H05C	109.5
C022—C028—C046	120.12 (18)	C057—C056—H05D	109.5
C022—C028—H028	119.9	H05C—C056—H05D	109.5
C046—C028—H028	119.9	C057—C056—H05E	109.5
C043—C029—C040	117.31 (16)	H05C—C056—H05E	109.5
C043—C029—H02A	108	H05D—C056—H05E	109.5
C040—C029—H02A	108	O012—C057—C056	110.4 (2)
C043—C029—H02B	108	O012—C057—H05F	109.6
C040—C029—H02B	108	C056—C057—H05F	109.6
H02A—C029—H02B	107.2	O012—C057—H05G	109.6
C017—C030—P002	111.60 (12)	C056—C057—H05G	109.6
C017—C030—H03A	109.3	H05F—C057—H05G	108.1
P002—C030—H03A	109.3	C055—C058—H05H	109.5
C017—C030—H03B	109.3	C055—C058—H05I	109.5
P002—C030—H03B	109.3	H05H—C058—H05I	109.5
H03A—C030—H03B	108	C055—C058—H05J	109.5
C044—C031—C037	120.8 (2)	H05H—C058—H05J	109.5
C044—C031—H031	119.6	H05I—C058—H05J	109.5
C037—C031—H031	119.6		
			
P003—Cu01—P002—C027	119.57 (7)	Cu01—P002—C027—C038	120.60 (17)
P004—Cu01—P002—C027	−60.79 (7)	C013—C022—C028—C046	−0.3 (3)
P003—Cu01—P002—C030	−122.85 (7)	C017^i^—C017—C030—P002	179.00 (16)
P004—Cu01—P002—C030	56.79 (7)	C027—P002—C030—C017	173.01 (13)
P003—Cu01—P002—C014	−1.56 (7)	C014—P002—C030—C017	−77.86 (14)
P004—Cu01—P002—C014	178.07 (6)	Cu01—P002—C030—C017	51.64 (14)
P002—Cu01—P003—C020	−79.86 (7)	C020—P003—C032—C040	−62.16 (15)
P004—Cu01—P003—C020	100.50 (7)	C024—P003—C032—C040	−174.22 (13)
P002—Cu01—P003—C024	42.64 (8)	Cu01—P003—C032—C040	56.76 (14)
P004—Cu01—P003—C024	−137.00 (7)	C015—C021—C033—C045	0.2 (3)
P002—Cu01—P003—C032	164.55 (7)	P004—C021—C033—C045	−178.67 (16)
P004—Cu01—P003—C032	−15.09 (7)	C047—C034—C035—C049	1.4 (4)
P002—Cu01—P004—C021	88.21 (7)	C013—C014—C036—C046	−0.5 (3)
P003—Cu01—P004—C021	−92.08 (7)	P002—C014—C036—C046	−179.15 (14)
P002—Cu01—P004—C039	−31.21 (8)	C044—C031—C037—C024	0.3 (3)
P003—Cu01—P004—C039	148.50 (7)	C016—C024—C037—C031	−0.3 (3)
P002—Cu01—P004—C043	−148.56 (7)	P003—C024—C037—C031	178.27 (16)
P003—Cu01—P004—C043	31.15 (7)	C047—C027—C038—C049	0.8 (3)
C022—C013—C014—C036	0.8 (3)	P002—C027—C038—C049	−176.02 (19)
C022—C013—C014—P002	179.38 (15)	C041—C019—C039—C052	0.2 (3)
C027—P002—C014—C036	−137.70 (15)	C041—C019—C039—P004	171.72 (15)
C030—P002—C014—C036	112.59 (15)	C021—P004—C039—C052	−37.54 (18)
Cu01—P002—C014—C036	−12.95 (17)	C043—P004—C039—C052	−147.75 (16)
C027—P002—C014—C013	43.71 (17)	Cu01—P004—C039—C052	88.38 (16)
C030—P002—C014—C013	−66.00 (16)	C021—P004—C039—C019	151.01 (15)
Cu01—P002—C014—C013	168.45 (12)	C043—P004—C039—C019	40.79 (17)
C042—C018—C020—C023	0.1 (3)	Cu01—P004—C039—C019	−83.08 (16)
C042—C018—C020—P003	179.00 (16)	C043—C029—C040—C032	56.8 (2)
C024—P003—C020—C018	82.72 (18)	P003—C032—C040—C029	−116.84 (16)
C032—P003—C020—C018	−28.92 (19)	C050—C026—C041—C019	0.8 (3)
Cu01—P003—C020—C018	−148.02 (15)	C039—C019—C041—C026	−0.8 (3)
C024—P003—C020—C023	−98.42 (16)	C020—C018—C042—C053	0.3 (3)
C032—P003—C020—C023	149.94 (16)	C040—C029—C043—P004	48.1 (2)
Cu01—P003—C020—C023	30.85 (17)	C021—P004—C043—C029	52.85 (16)
C051—C015—C021—C033	0.2 (3)	C039—P004—C043—C029	162.59 (14)
C051—C015—C021—P004	179.04 (19)	Cu01—P004—C043—C029	−75.88 (14)
C039—P004—C021—C015	121.03 (17)	C037—C031—C044—C025	0.5 (3)
C043—P004—C021—C015	−129.92 (17)	C016—C025—C044—C031	−1.2 (3)
Cu01—P004—C021—C015	−1.88 (19)	C021—C033—C045—C048	−0.5 (3)
C039—P004—C021—C033	−60.12 (18)	C022—C028—C046—C036	0.6 (3)
C043—P004—C021—C033	48.93 (19)	C014—C036—C046—C028	−0.1 (3)
Cu01—P004—C021—C033	176.96 (14)	C035—C034—C047—C027	0.2 (3)
C014—C013—C022—C028	−0.4 (3)	C038—C027—C047—C034	−1.2 (3)
C018—C020—C023—C054	−0.2 (3)	P002—C027—C047—C034	175.70 (17)
P003—C020—C023—C054	−179.12 (17)	C033—C045—C048—C051	0.4 (4)
C025—C016—C024—C037	−0.3 (3)	C034—C035—C049—C038	−1.9 (4)
C025—C016—C024—P003	−179.02 (15)	C027—C038—C049—C035	0.8 (4)
C020—P003—C024—C037	−17.76 (19)	C041—C026—C050—C052	−0.3 (3)
C032—P003—C024—C037	93.35 (17)	C045—C048—C051—C015	0.0 (4)
Cu01—P003—C024—C037	−142.28 (14)	C021—C015—C051—C048	−0.2 (4)
C020—P003—C024—C016	160.86 (15)	C019—C039—C052—C050	0.3 (3)
C032—P003—C024—C016	−88.03 (16)	P004—C039—C052—C050	−171.28 (16)
Cu01—P003—C024—C016	36.34 (16)	C026—C050—C052—C039	−0.2 (3)
C024—C016—C025—C044	1.1 (3)	C018—C042—C053—C054	−0.8 (3)
C030—P002—C027—C047	−178.12 (15)	C042—C053—C054—C023	0.8 (4)
C014—P002—C027—C047	73.07 (17)	C020—C023—C054—C053	−0.3 (3)
Cu01—P002—C027—C047	−56.18 (17)	C057—O012—C055—C058	178.0 (2)
C030—P002—C027—C038	−1.3 (2)	C055—O012—C057—C056	−179.5 (2)
C014—P002—C027—C038	−110.14 (18)		

Symmetry code: (i) −*x*+1, −*y*, −*z*+1.

### Hydrogen-bond geometry (Å, º)

**Table:**

*D*—H···*A*	*D*—H	H···*A*	*D*···*A*	*D*—H···*A*
C016—H016···F006	0.95	2.47	3.318 (3)	149
C018—H018···F008^ii^	0.95	2.53	3.286 (3)	137
C043—H04*C*···F006	0.99	2.45	3.345 (3)	150
C043—H04*C*···F009	0.99	2.53	3.458 (3)	156
C049—H049···O012^i^	0.95	2.46	3.393 (3)	169

Symmetry codes: (i) −*x*+1, −*y*, −*z*+1; (ii) −*x*+2, −*y*+1, −*z*+1.
